# Infrared photovoltaic detector based on p-GeTe/n-Si heterojunction

**DOI:** 10.1186/s11671-020-03336-7

**Published:** 2020-06-29

**Authors:** Yiqun Zhao, Libin Tang, Shengyi Yang, Shu Ping Lau, Kar Seng Teng

**Affiliations:** 1 School of Physics, Beijing Institute of Technology, Beijing, 100081 China; 2 Kunming Institute of Physics, Kunming, 650223 China; 3 Yunnan Key Laboratory of Advanced Photoelectric Materials and Devices, Kunming, 650223 China; 4 Kunming Metallurgy College, Kunming, 650033 China; 5 Department of Applied Physics, The Hong Kong Polytechnic University, Hong Kong SAP, People’s Republic of China; 6 College of Engineering, Swansea University, Bay Campus, Fabian Way, Swansea, SA1 8EN UK

**Keywords:** GeTe, Heterojunction, Optoelectronic characteristics, Photovoltaic detector

## Abstract

GeTe is an important narrow bandgap semiconductor material and has found application in the fields of phase change storage as well as spintronics devices. However, it has not been studied for application in the field of infrared photovoltaic detectors working at room temperature. Herein, GeTe nanofilms were grown by magnetron sputtering technique and characterized to investigate its physical, electrical, and optical properties. A high-performance infrared photovoltaic detector based on GeTe/Si heterojunction with the detectivity of 8 × 10^11^ Jones at 850 nm light irradiation at room temperature was demonstrated.

## Background

There has been a great interest in infrared detectors due to its many potential applications in night vision imaging, safety, remote sensing, food inspection, biology, and other fields [1–3]. Generally, photovoltaic infrared detectors take advantage of minority carrier effects leading to short response time, which is ideal for imaging and sensing applications. HgCdTe-based infrared detector is well established [4, 5]. However, the lattice mismatch of HgCdTe and Si does not permit integration of detection and data processing units, hence resulting in costly system and hindering miniaturization of the technology.

There have been much research activities in developing various heterogeneous structures based on two-dimensional materials grown on different substrates [6–9]. The resultant heterogeneous structure depends on van der Waals interaction [10], and there is no requirement for lattice matching of the different materials.

GeTe material has attracted extensive attention in recent years [11–15]. It has been considered as a strong contender for the next-generation memory technology as the material exhibits different physical, electrical, and optical properties when it is in amorphous and crystal phases [16–21]. GeTe can also be made into dilute magnetic semiconductor, which is an important material for spintronics devices [15, 22, 23]. If the unique storage and computing features of GeTe can be integrated to develop novel devices, this will lead to significant advancement in the computing technology.

Furthermore, the ability to develop photovoltaic detector based on two-dimensional GeTe and Si heterojunction will lead to groundbreaking technology due to their compatibility with Si circuit and GeTe-based spintronic device processes. It will facilitate seamless and fast connection involving photovoltaic detectors in the field of computing in the future. Importantly, the technology is suitable for miniaturization at low cost.

In this work, p-type GeTe nanofilms were prepared by magnetron sputtering and annealing methods. The physical, electronic, and optical properties of the nanofilms were investigated. Finally, a photovoltaic detector based on p-GeTe/n-Si heterojunction was fabricated, and its performance was characterized.

## Methods

**Fig. 1:**
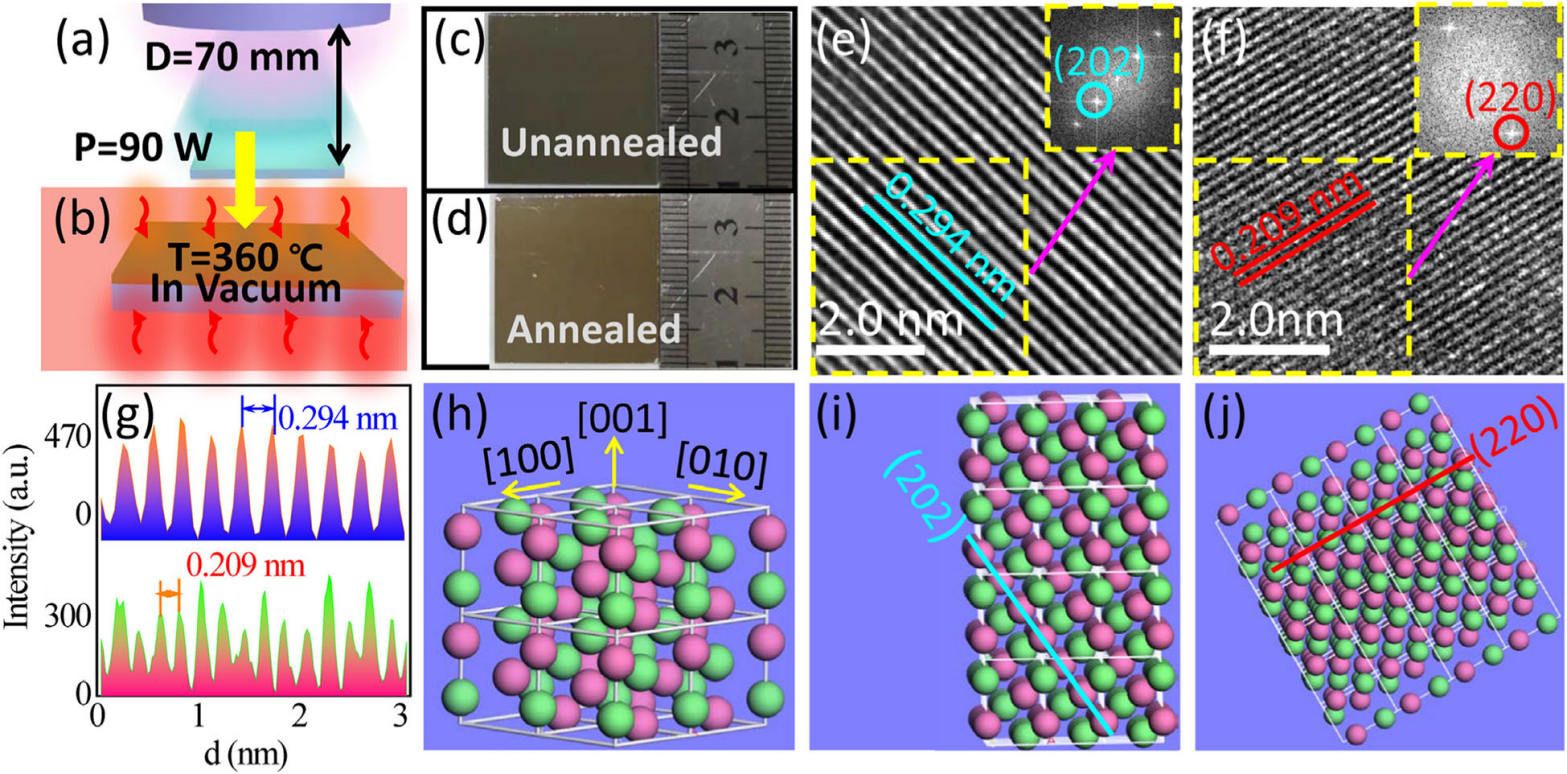
**a** Magnetron sputtering of GeTe film on Si substrate. **b** Post-annealing of the GeTe film. **c** Optical images of as-deposited and **d** annealed GeTe films on quartz substrate. **e**–**f** TEM images and FFT patterns (inset) of the annealed GeTe film. **g** Line profiles of the lattice fringes of (202) and (220) crystal planes as shown in the top and bottom panels, respectively. **h**–**j** Schematic diagrams of the crystal structures

The device was fabricated using the following processes. First, an n-type monocrystalline silicon (Si) substrate was cleaned by chemical bath method using a mixed solution containing H_2_O to H_2_O_2_ to NH_3_∙H_2_O (3:1:1) at 80 °C for 30 min and dried under air flow. GeTe film was then deposited by magnetron sputtering directly onto the cleaned substrate at a pressure of 5 Pa for 120 s from an initial vacuum of 6.0 × 10^−4^ Pa. Subsequently, the film was wrapped in copper foil and then annealed in a vacuum oven at 360 °C for 10 min. The annealing method was based on preliminary experiments and previously reported phase transition temperature of the material in the literatures [18, 24–26]. Finally, a pair of aluminum (Al) electrodes was evaporated onto the GeTe film and Si substrate using physical vapor deposition (PVD) technique (at a pressure of 7.0 × 10^−5^ Pa) through a shadow mask. The thickness of the Al electrodes was approximately 100 nm as measured by a quartz oscillator during deposition. The effective area of the device was 1.5 mm^2^. Figure 1a and b illustrate the magnetron sputtering and oven annealing processes, respectively. Figure 1c and d show the as-deposited and annealed GeTe films, respectively.

## Results and Discussion

High-resolution transmission electron microscopy (HRTEM) images of the annealed GeTe film are shown in Fig. 1 e and f. The insets show the fast Fourier transform (FFT) patterns of the GeTe film. Indices of crystal planes are indicated on the images. According to these results, the annealed GeTe film exhibited good crystallinity. Figure 1g shows the line profiles of the lattice fringes shown in Fig. 1e and f. The top and bottom line profiles of Fig. 1g corresponds to (202) and (220) crystal planes of GeTe film, which has a lattice fringe separation of 0.294 and 0.209 nm, respectively. Schematic diagram of the GeTe lattice structure is illustrated in Fig. 1h. Figure 1i and j show crystal plane models of GeTe as observed in Fig. 1e and f, respectively.

**Fig. 2:**
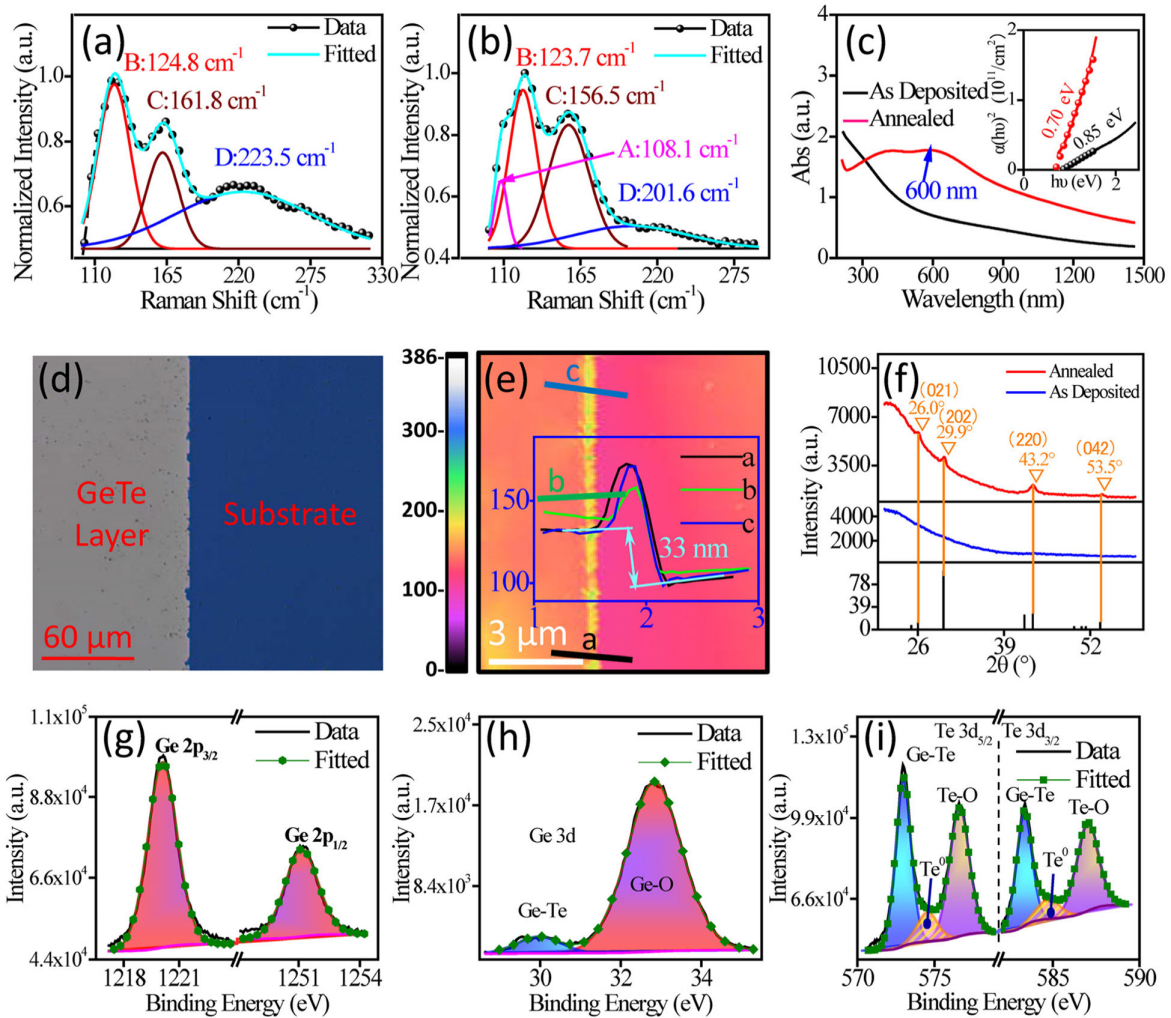
**a**–**b** Normalized Raman spectra of GeTe films before and after annealing, respectively. **c** UV-Vis-NIR absorption spectra of the GeTe films before and after annealing. (Inset) Plot of *α*^*2*^ versus photon energy (*hν*) of the two GeTe films. **d** Optical images of the annealed GeTe film for AFM measurement. **e** AFM image and line profiles (inset) for thickness measurement of the annealed GeTe film. **f** XRD spectra of the GeTe films before and after annealing. **g**–**i** XPS spectra of Ge 2p, Ge 3d, and Te 3d core levels of the annealed GeTe film

Raman spectroscopy was performed to study the structure of the GeTe films before and after annealing using a Renishaw inVia Raman microscope equipped with an argon-ion laser operating at an excitation wavelength of 514 nm. Figure 2a and b show the normalized Raman spectra of as-deposited and annealed GeTe films, respectively. The results are in good agreement with the literatures [27, 28]. There were three distinctive bands between 100 and 300 cm^−1^ as shown in Fig. 2a. These bands were situated at 124.8, 161.8, and 223.5 cm^−1^, namely, bands B, C, and D, respectively. After annealing, there was a significant reduction in band D and also an appearance of band A situated at 108.1 cm^−1^ as shown in Fig. 2b. Bands B, C, and D were also red-shifted by 1.1, 5.3, and 21.9 cm^−1^, respectively. These are attributed to structural transformation of the GeTe film resulting in reduction in the degree of disorder (e.g., ratio of intermolecular to intramolecular interactions) [27].

To investigate the optical properties of the GeTe films before and after annealing, UV-Vis-NIR absorption spectroscopy was performed using a Horiba iHR 320 spectrometer. Figure 2c shows the UV-Vis-NIR absorption spectra obtained from both films. An absorption peak at 600 nm was apparent after annealing. The absorption coefficient of annealed GeTe film was significantly larger than that of unannealed film. Furthermore, a decreasing trend in the absorption coefficient was observed for an increasing wavelength in the infrared band. Bandgap energy (*E*_*g*_) of the films can be determined using the following formulae [29, 30]:

1 $$ {\alpha}^2\left( h\nu \right)=C\left( h\nu -{E}_g\right) $$

where *hν* is the energy of incident photon, *α* is the optical absorption coefficient associated with *hν*, and *C* is a constant. The direct optical bandgap of the GeTe films can be estimated from the curve of *α*^*2*^ vs. photon energy (*hv*) as shown in the inset of Fig. 2c. It can vary greatly depending on the experimental conditions and theoretical models [31]. In this work, the estimated *E*_*g*_ of the GeTe films before and after annealing was 0.85 and 0.70 eV, respectively. This is in good agreement with previous work performed by others, which reported an optical bandgap of ~ 0.85 eV for an amorphous GeTe film and ~ 0.73–0.95 eV for crystalline film [32]. A reduction of *E*_*g*_ was reported after annealing because of long-range ordering of the lattice.

Atomic force microscopy (AFM) was carried out to determine the thickness of the films using AFM (SPA-400). Photoresist mask was used to prepare the sample for AFM measurements. Figure 2d shows an optical image of the prepared sample for AFM with an obvious boundary between GeTe film and substrate. Figure 2e reveals a film thickness of 33 ± 1.5 nm on Si substrates after annealing. Annealing has a little effect on the root-mean-square (RMS) surface roughness of the GeTe thin films; the RMS surface roughness decreased from 2.1 nm (as-deposited GeTe) to 1.4 nm (annealed GeTe).

The effect of annealing on the structure of GeTe nanofilms was further investigated using X-ray diffraction (XRD). Figure 2f shows the XRD spectra of the as-deposited (blue) and annealed (red) GeTe nanofilms. Two strong diffraction peaks at 29.9° and 43.2°, which corresponded to (202) and (220) lattice planes respectively, appeared after annealing. In addition, two weak diffraction peaks at 26.0° and 53.5°, which corresponded to (021) and (042) lattice planes respectively, also appeared in the spectrum. When combined with the above TEM results, it is evident that the GeTe nanofilm preferentially ordered along (220) and (202) lattice planes during the annealing process. Compared to the as-deposited GeTe films, the annealed GeTe has a drastic change in the crystal phase; the difference in the structure-related optical properties (absorption spectra) is shown in Fig. 2f and c.

Elemental composition and chemical bonds at the surface of annealed GeTe nanofilms were studied by X-ray photoelectron spectroscopy (XPS) using AlKα radiation with energy of 1486.6 eV. XPS spectra of Ge 2p, Ge 3d and Te 3d core level peaks of the annealed GeTe film are shown in Fig. 2g, h and i, respectively. The Ge 2p core level consisted mainly of Ge 2p_3/2_ (1220.1 eV) and Ge 2p_1/2_ (1251.1 eV) doublet peaks. The Ge 3d core level was deconvoluted into two components, namely Ge-Te and Ge-O at binding energy of 30.0 and 32.8 eV, respectively. The Te 3d core level consisted of Ge-Te, Te-O and Te-Te components. The Te-O (Te^4+^) peaks at 576.5 eV (Te 3d_5/2_) and 587.0 eV (Te 3d_3/2_) in Fig. 2i were associated with TeO_2_ [33, 34]. Both Ge 3d and Te 3d core levels of annealed GeTe nanofilm exhibited oxygen-related components as shown in Fig. 2h and i, respectively. However, there was no oxygen-related component at Ge 2p core level, which was at greater penetration depth, as shown in Fig. 2g. Furthermore, GeO_2_ and TeO_2_ were absent from the XRD and TEM characterizations, hence this suggests that the oxidation of Ge and Te atoms were primarily localized at the surface of the film by atmospheric oxygen during the transfer and annealing processes [34] and the oxide layer was very thin. In addition, the annealed GeTe films were investigated by Hall measurement which revealed the p-type conductance.

**Fig. 3:**
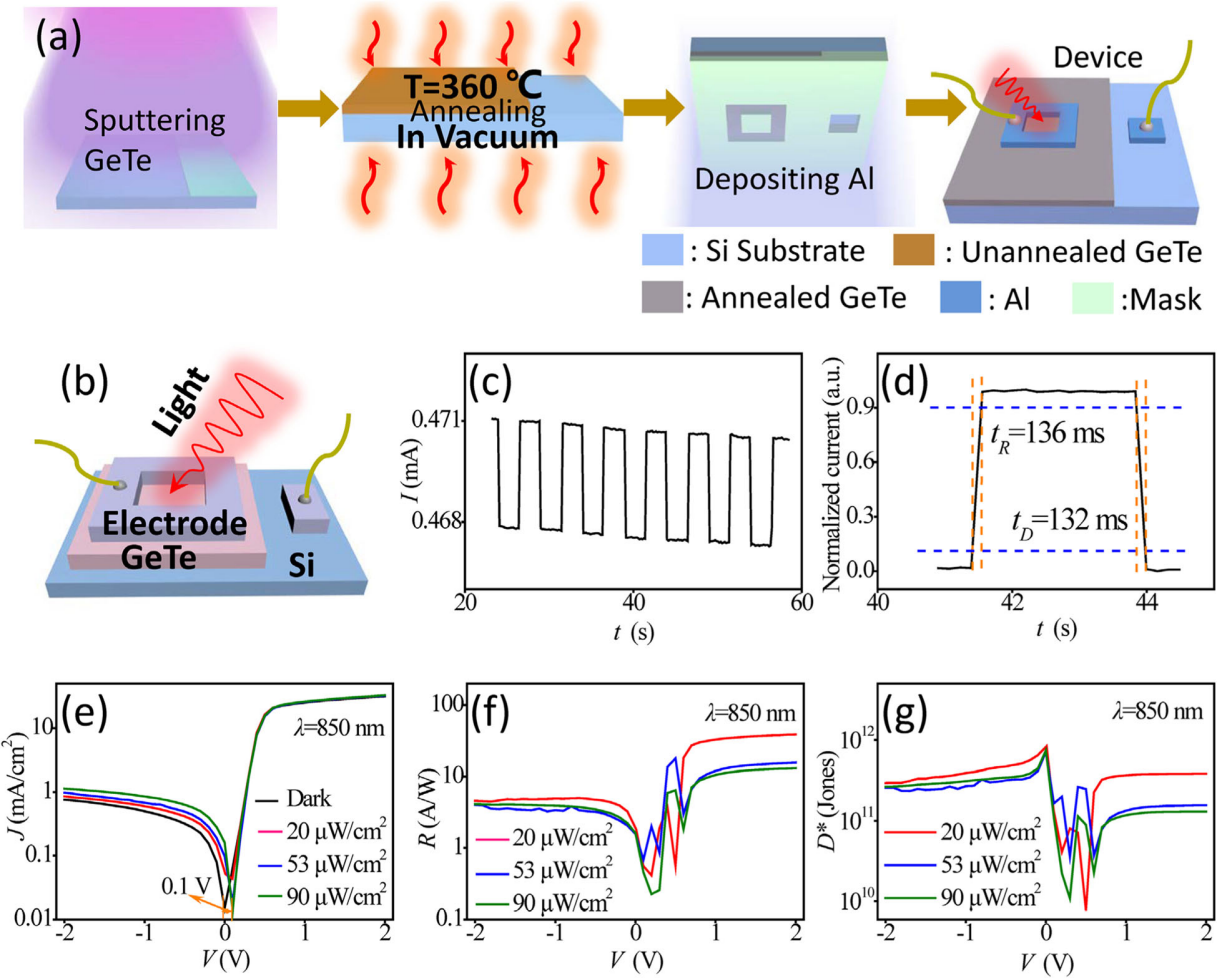
**a** Schematic diagrams illustrating the fabrication process of photovoltaic detector based on p-GeTe/n-Si heterojunction and **b** the device structure. **c**–**d** Temporal photoresponse of the device. **e** Plots of log(*J*)*-V* characteristics of the photovoltaic detector under dark (black line) and different irradiation densities (colored lines). **f** Plots of *R* (responsivity)-*V* and **g**D^*^ (detectivity)-*V* characteristics of the photovoltaic detector

A prototype photovoltaic detector based on p-GeTe/n-Si heterojunction was fabricated to explore the use of the material in the field of optoelectronics. The device fabrication processes are illustrated in Fig. 3a. Figure 3b depicts the structure of the photodetector. The thickness of the GeTe film and Al electrodes was 33 and 100 nm, respectively. Figure 3c and d show the response time of the device. The rise time (*t*_R_) is defined as time taken for the current to increase from 10 to 90% of the peak, while the decay time (*t*_D_) is time taken for current to decrease from 90 to 10%. As shown, the rise and fall time were symmetrical with a response time (τ) of 134 ms (e.g., (t_R_ + t_D_)/2).

Photoresponse of the device was evaluated from *J*-*V* measurements using Keithley 2400 sourcemeter under light illumination. The log *J* vs. *V* characteristics of the device irradiated by *λ* = 850 nm light at different densities of 20, 53, and 90 μW cm^−2^ and under dark condition performed at room temperature are shown in Fig. 3e. It can be seen from Fig. 3e that the voltage corresponding to the minimum value of *J*_*opt*_ (i.e., photocurrent density) deviated by 0.1 V from the voltage corresponding to the minimum value of *J*_*D*_ (i.e., dark current density) in the direction of positive bias, and that the photogenic voltage was generated under the light conditions. Therefore, the p-GeTe/n-Si heterojunction has demonstrated its potential application in infrared detection.

Two important figures of merit for photodetector, such as responsivity (*R*) and detectivity (D^*^), were determined using the following equations [35, 36]:

2 $$ R=\frac{I_p}{AP_{opt}} $$

3 $$ {D}^{\ast }=\frac{R\sqrt{A}}{\sqrt{2q\left|{I}_d\right|}} $$

where *I*_*p*_ is the photocurrent that equals to absolute value of current under irradiation subtracting that in the dark, *A* is the effective area of the device, *P*_*opt*_ is the incident optical power, *I*_*d*_ is the dark current, and *q* is the unit charge (1.6 × 10^−19^ C).

The values of *R* and D^*^ were 6–15 A/W and 1–8 × 10^11^ Jones (1 Jones = 1 cm Hz^1/2^ W^−1^) as obtained from Fig. 3f and g, respectively. The device was evaluated at room temperature, unpackaged, and without optimization. Table 1 lists the responsivity and detectivity of some infrared photodetectors based chalcogenide/Si heterojunction; it can be seen that GeTe/Si shows a relatively higher performance at room temperature, which maybe due to the big absorption coefficient and the direct band gap of GeTe.

**Table 1:** Comparison of responsivity and detectivity of infrared photodetectors based on other materials forming heterojunction with Si

Heterojunction	Wavelength (nm)	***R*** (A/W)	D* (Jones)	Ref.
GeTe/Si	850	6 - 15	1–8 × 10^11^	This work
Bi_2_Se_3_/Si	808	24.28	4.4 × 10^12^	[37]
SnS/Si	850	0.083	5.3 × 10^9^	[38]
MoS_2_/Si	300–1100	0.0119	2.1 × 10^10^	[39]
WS_2_/Si	400–1100	1.11	5 × 10^11^	[40]
WS_2_/Si	Near infrared	3.7–4.5		[41]

## Conclusions

Crystalline GeTe nanofilms were produced by magnetron sputtering and post-annealing treatment. The physical, electronic, and optical properties of the nanofilms before and after annealing were studied. After annealing at 360 °C, the nanofilm revealed long-range order and bandgap energy of 0.70 eV. Photovoltaic detector based on the p-GeTe/n-Si heterojunction was fabricated and demonstrated photoresponse at 850 nm irradiation exhibiting high *R* of 6–15 A/W and D* of 1–8 × 10^11^ Jones with a response time of 134 ms. Hence, the use of p-GeTe/n-Si heterojunction in infrared detection was demonstrated in this work. It has enormous potential for integration with other fields, such as computing and data storage.

## Data Availability

The conclusions made in this manuscript are based on the data (main text and figures) presented and shown in this paper.

## Abbreviations

PVD: Physical vapor deposition
TEM: Transmission electron microscope
HRTEM: High-resolution transmission electron microscope
FFT: Fast Fourier transform
AFM: Atomic force microscope
XRD: X-ray diffractometer
XPS: X-ray photoelectron spectroscopy

## References

[1] Abbas MM, Kostiuk T, Ogilvie KW (1976). Infrared upconversion for astronomical applications. Appl Opt.

[2] Tan MC, Al-Baroudi L, Riman RE (2011). Surfactant effects on efficiency enhancement of infrared-to-visible upconversion emissions of NaYF_4_:Yb-Er. ACS Appl Mater Interfaces.

[3] Ring EF, Ammer K (2012). Infrared thermal imaging in medicine. Physiol Meas.

[4] Rogalski A, Martyniuk P, Kopytko M (2019) Type-II superlattice photodetectors versus HgCdTe photodiodes. Progr Quantum Electron 100228

[5] Kopytko M, Rogalski A (2016). HgCdTe barrier infrared detectors. Progr Quantum Electron.

[6] Gan X, Shiue R-J, Gao Y (2013). Chip-integrated ultrafast graphene photodetector with high responsivity. Nat Photonics.

[7] Wang X, Cheng Z, Xu K (2013). High-responsivity graphene/silicon-heterostructure waveguide photodetectors. Nat Photonics.

[8] Gan X, Gao Y, Fai Mak K (2013). Controlling the spontaneous emission rate of monolayer MoS_2_ in a photonic crystal nanocavity. Appl Phys Lett.

[9] Sobhani A, Lauchner A, Najmaei S (2014). Enhancing the photocurrent and photoluminescence of single crystal monolayer MoS_2_ with resonant plasmonic nanoshells. Appl Phys Lett.

[10] Wang L, Meric I, Huang PY (2013). One-dimensional electrical contact to a two-dimensional material. Science.

[11] Zhang D, Zhou Z, Wang H et al (2018) Tunable electric properties of bilayer α-GeTe with different interlayer distances and external electric fields. Nanoscale Res Lett 13 10.1186/s11671-018-2813-x PMC6286292 30536206

[12] Kolobov AV, Krbal M, Fons P (2011). Distortion-triggered loss of long-range order in solids with bonding energy hierarchy. Nat Chem.

[13] Zhang J, Huang R, Shi L (2013). Bi doping modulating structure and phase-change properties of GeTe nanowires. Appl Phys Lett.

[14] Wang L, Yang CH, Wen J (2016). Overview of probe-based storage technologies. Nanoscale Res Lett.

[15] Hoffmann A, Bader SD (2015). Opportunities at the frontiers of spintronics. Phys Rev Applied.

[16] Kooi BJ, Momand J (2019). High resolution imaging of chalcogenide superlattices for data storage applications: progress and prospects. Physica Status Solidi (RRL) – Rapid Research Letters.

[17] Meena J, Sze S, Chand U (2014). Overview of emerging nonvolatile memory technologies. Nanoscale Res Lett.

[18] Siegrist T, Jost P, Volker H (2011). Disorder-induced localization in crystalline phase-change materials. Nat Mater.

[19] Zhang W, Mazzarello R, Wuttig M (2019). Designing crystallization in phase-change materials for universal memory and neuro-inspired computing. Nat Rev Mater.

[20] Wuttig M, Yamada N (2007). Phase-change materials for rewriteable data storage. Nat Mater.

[21] Wang L, Lu S-R, Wen J (2017). Recent advances on neuromorphic systems using phase-change materials. Nanoscale Res Lett.

[22] Rinaldi C, Varotto S, Asa M (2018). Ferroelectric control of the spin texture in GeTe. Nano Lett.

[23] Manchon A, Koo HC, Nitta J (2015). New perspectives for Rashba spin-orbit coupling. Nat Mater.

[24] Ren K, Zhu M, Song W (2019). Electrical switching properties and structural characteristics of GeSe-GeTe films. Nanoscale.

[25] Carria E, Mio AM, Gibilisco S (2011). Amorphous-crystal phase transitions in Ge_x_Te_1-x_ alloys. J Electrochem Soc.

[26] Chua EK, Shi LP, Zhao R (2010). Low resistance, high dynamic range reconfigurable phase change switch for radio frequency applications. Appl Phys Lett.

[27] Andrikopoulos KS, Yannopoulos SN, Voyiatzis GA (2006). Raman scattering study of the a-GeTe structure and possible mechanism for the amorphous to crystal transition. J Phys Condens Matter.

[28] Sarkar D, Sanjeev G, Mahesha MG (2015). Analysis of electron beam-induced effect on electrical switching properties of glass chalcogenide GeTe thin films through Raman spectroscopy. Appl Phys A Mater Sci Process.

[29] Manser JS, Christians JA, Kamat PV (2016). Intriguing optoelectronic properties of metal halide perovskites. Chem Rev.

[30] Tauc J, Menth A (1972). States in the gap. J Non-Cryst Solids.

[31] Vadkhiya L, Arora G, Rathor A (2011). Electron momentum density and band structure calculations of α- and β-GeTe. Radiat Phys Chem.

[32] Bahl SK, Chopra KL (1970). Amorphous versus crystalline GeTe films. III. Electrical Properties and Band Structure. J Appl Phys.

[33] Qian H, Tong H, Zhou LJ (2016). Low work function of crystalline GeTe/Sb_2_Te_3_ superlattice-like films induced by Te dangling bonds. J Phys D Appl Phys.

[34] Yashina LV, Kobeleva SP, Shatalova TB (2001). XPS study of fresh and oxidized GeTe and (Ge,Sn)Te surface. Solid State Ionics Diff React.

[35] Yang Y, Dai H, Yang F (2019). All-perovskite photodetector with fast response. Nanoscale Res Lett.

[36] Tan H, Fan C, Ma L (2015). Single-crystalline InGaAs nanowires for room-temperature high-performance near-infrared photodetectors. Nano Micro Lett.

[37] Zhang H, Zhang X, Liu C (2016). High-responsivity, high-detectivity, ultrafast topological insulator Bi_2_Se_3_/silicon heterostructure broadband photodetectors. ACS Nano.

[38] Patel M, Kim H-S, Kim J (2017). Wafer-scale production of vertical SnS multilayers for high-performing photoelectric devices. Nanoscale.

[39] Zhang Y, Yu Y, Mi L (2016). In situ fabrication of vertical multilayered MoS_2_/Si homotype heterojunction for high-speed visible-near-infrared photodetectors. Small.

[40] Chowdhury RK, Maiti R, Ghorai A (2016). Novel silicon compatible p-WS_2_ 2D/3D heterojunction devices exhibiting broadband photoresponse and superior detectivity. Nanoscale.

[41] Lan C, Li C, Wang S (2016). Zener tunneling and photoresponse of a WS_2_/Si van der Waals heterojunction. ACS Appl Mater Interfaces.

